# Two Novel Mutations in the *SI* Gene Associated With Congenital Sucrase-Isomaltase Deficiency: A Case Report in China

**DOI:** 10.3389/fped.2021.731716

**Published:** 2021-12-02

**Authors:** Jianli Zhou, Yuzhen Zhao, Xia Qian, Yongwei Cheng, Huabo Cai, Moxian Chen, Shaoming Zhou

**Affiliations:** ^1^Division of Gastroenterology, Shenzhen Children's Hospital, Shenzhen, China; ^2^Key Laboratory of National Forestry and Grassland Administration on Subtropical Forest Biodiversity Conservation, College of Biology and the Environment, Nanjing Forestry University, Nanjing, China

**Keywords:** congenital, sucrase-isomaltase deficiency, mutation, gene, case report

## Abstract

**Background:** Congenital sucrase-isomaltase deficiency (CSID) is an autosomal recessive inherited disease that leads to the maldigestion of disaccharides and is associated with mutation of the sucrase-isomaltase (*SI*) gene. Cases of CSID are not very prevalent in China or worldwide but are gradually being identified and reported.

**Case Presentation:** We report a case involving a 14-month-old male who presented with failure to thrive that had begun after food diversification and was admitted for chronic diarrhea. We used a whole-exome sequencing (WES) approach to identify mutations in this patient's genome. WES revealed two novel heterozygous mutations in the *SI* gene, c.2626C > T (p.Q876^*^) and c.2872C > T (p.R958C), which were confirmed by Sanger DNA sequencing. With a strict sucrose- and starch-restricted diet, the patient's diarrhea was resolved, and he began to gain weight.

**Conclusions:** We report a case of novel variants in the *SI* gene that caused CSID. This report provides valuable information for the clinical field, especially in China.

## Introduction

Congenital sucrase-isomaltase deficiency (CSID, OMIM #222900) was first reported in 1960 by Weijers et al. (1). This deficiency has been defined as an autosomal recessive disease that is characterized by loss of sucrase or sucrase-isomaltase (*SI*) activities (2). Upon the ingestion of disaccharides and oligosaccharides, osmotic-fermentative diarrhea occurs due to the failure of sucrose breakdown into fructose and glucose. As a result, patients with CSID have chronic diarrhea, abdominal pain, and abdominal distension, leading to failure to thrive. The estimated prevalence of CSID in North America and Europe ranges from 0.05 to 0.2% (3], while in the Inuit population of Greenland, it ranges from 5 to 10% (4). However, the prevalence of CSID in the Chinese population is unknown (5).

Currently, the confirmation of complete or near-complete absence of sucrase and/or isomaltase activities in biopsy tissue from the small bowel is the diagnostic gold standard for CSID (6). This approach is straightforward but invasive and is difficult to implement in young patients. Lifelong sucrose restriction is an effective therapy for CSID patients (7).

At the genetic level, this condition results from compound heterozygous or homozygous mutations in the sucrase-isomaltase gene (*SI*, OMIM #609845), which is located on chromosome 3q26.1 (7). This locus encodes a small intestine brush-border membrane disaccharidase that is required for the hydrolysis of some starches and sucrose. The first identification of a mutation in the *SI* gene associated with CSID was described by Ouwendijk et al. (8). Previous reports have shown associations of *SI* mutations with irritable bowel syndrome (9). Genetic testing of the *SI* gene for this condition is now clinically available. To date, more than 40 mutations of the *SI* gene that are associated with CSID have been identified (10). Here, we report a case of CSID with two novel variants of the *SI* gene.

## Case Presentation

The patient was a 14-month-old male admitted to our hospital for chronic diarrhea, abdominal distention, and failure to thrive. He was the second child of healthy and non-consanguineous parents. Both his father and brother had a history of frequent episodes of diarrhea in their youth.

The patient was born at 39 weeks with a height of 50 cm, and he weighed approximately 3 kg (between the age- and sex-specific 15–25th percentile). He was breastfed at birth and showed ordinary growth until 3 months old (weight: 6 kg, at the age- and sex-specific 15–25th percentile), at which time food diversification began with goat milk, rice paste, and so on. After this dietary change for several days, he began to have seven to eight episodes per day of non-bloody watery stools and had poor weight gain. Formula changes, including deep hydrolysis formulas and amino acid formulas, were attempted without the patient showing signs of improvement. He was admitted to more than three hospitals at the ages of 6 months, 9 months, and 12 months, but treatment for a cow's milk protein allergy did not work.

A physical examination in our hospital suggested an alert infant with a severe reduction in subcutaneous fat. He weighed 5.6 kg (below the age- and sex-specific 3rd percentile) and had not gained weight for 11 months. He could not sit, crawl, or walk by himself.

**Figure 1:**
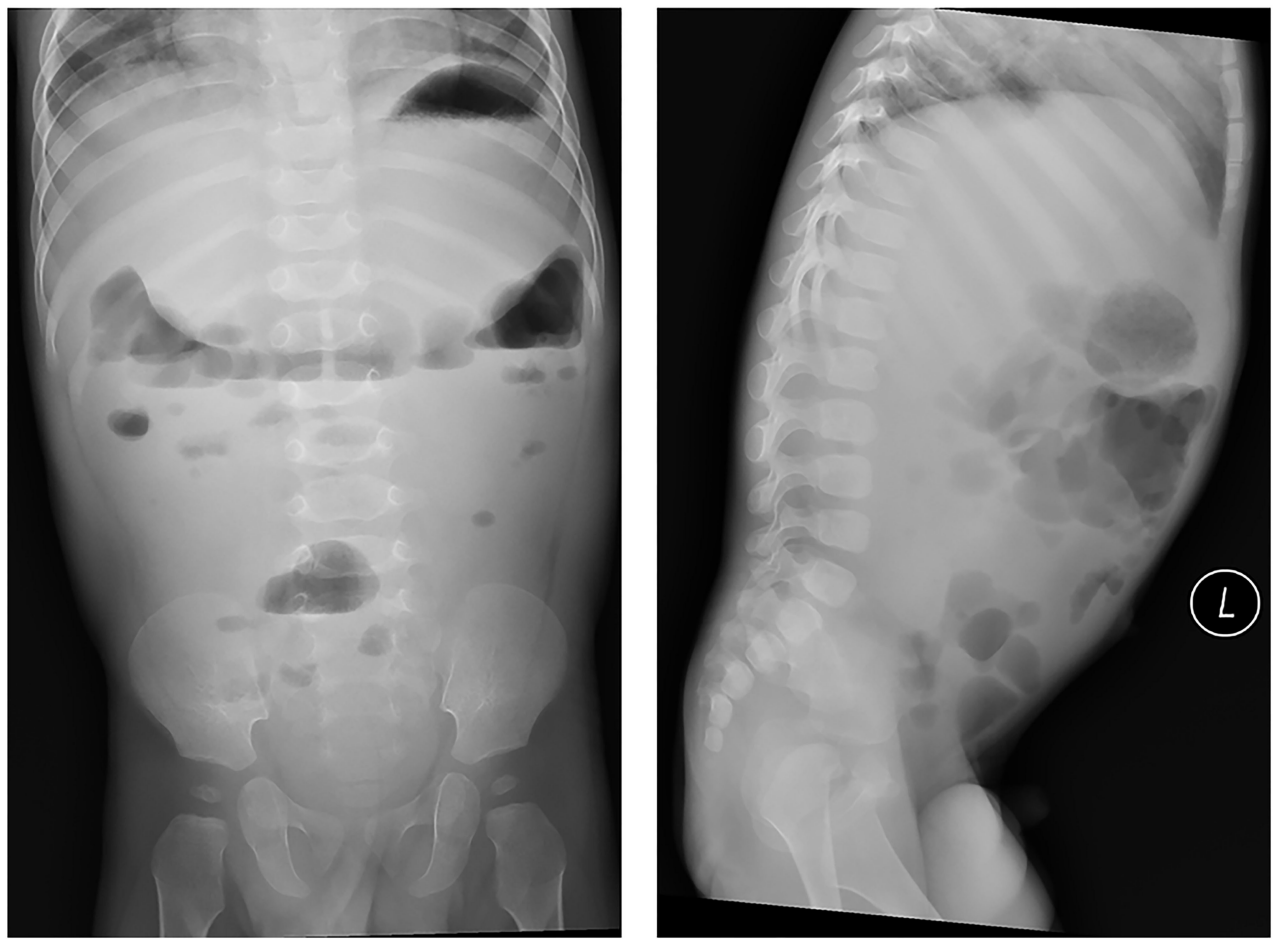
Abdominal radiographs suggested intestinal motility changes without any signs of intestinal obstruction.

The results of a laboratory examination showed that the patient's blood count levels, C-reactive protein levels, liver function, renal function and other blood or stool tests were normal. Imageology examination that included abdominal radiographs suggested intestinal motility changes without any signs of intestinal obstruction (Figure 1).

Although his parents did not consent to an invasive biopsy of small bowel tissue, they agreed to whole-exome sequencing (WES) to identify their son's underlying genetic mutations. A genetic study was carried out after approval from the Clinical Research Ethics Committee. Informed consent was obtained. His parents also received genetic testing.

**Figure 2:**
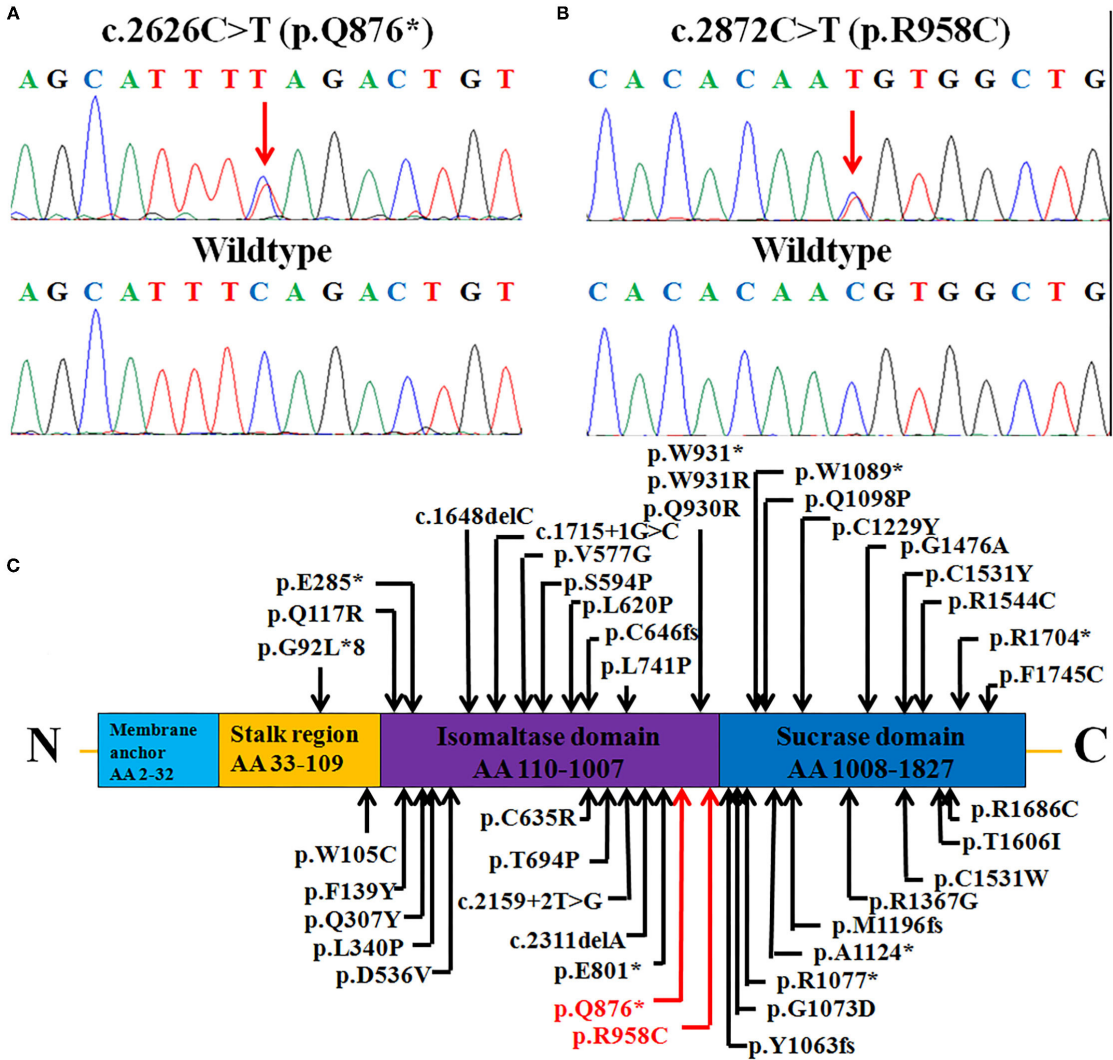
Heterozygous *SI* mutations: **(A)** A non-sense mutation c.2626C > T (p.Q876*) and **(B)** missense mutation c.2872C > T (p.R958C) were identified in the patient (upper panels), while healthy control individuals had the wild-type sequence (lower panels). **(C)** The structure of the *SI* protein (NP_001032.2), depicting the functional domains, 40 reported mutations, and two mutations in this case. The mutations identified in this study are marked in red (novel), and previously reported mutations are marked in black. AA, amino acids.

As a result, two novel heterozygous mutations, c.2626C > T (p.Q876^*^) (inherited from his father) and c.2872C > T (p.R958C) (inherited from his mother), in the *SI* gene were identified and confirmed by Sanger sequencing (Figures 2A,B), leading to the diagnosis of CSID.

This patient was given a strict sucrose- and starch-restricted diet. Without sucrose and starch, the patient's diarrhea resolved. Follow-up revealed that the patient grew and that gradually caught up in weight with other children his age. Three months later (at 17 months old), he weighed 7.6 kg (below the age- and sex-specific 3rd percentile); 1 year later (at 2 years and 2 months old), he weighed 10.5 kg (below the age- and sex-specific 3rd percentile); and 3 years later (at 4 years and 2 months old), he weighed 15 kg (at the age- and sex-specific 15–25th percentile) and was 104 cm tall (at the age- and sex-specific 25–50th percentile). Furthermore, he had learned to sit, crawl, walk, and run (gradually catching up with other children his age), which he could not do before. These observations suggested that our treatment was effective.

## Discussion and Conclusion

CSID is an inherited disease that occurs due to pathogenic variants of the *SI* gene (7). The phenotypes of patients are heterogeneous and vary according to onset age. Patients with an onset of CSID in infancy typically present symptoms that include diarrhea and failure to thrive, as observed in our case. Patients with an onset of CSID in childhood or adulthood present milder symptoms with only chronic diarrhea and have normal growth rates (11). According to a summary of previously reported cases (2, 6–8, 11–21), the incidence of CSID differs between females and males (19/60) (Table 1). However, in some of these cases, sex was not reported. The patients had diarrhea (29/29) and failure to thrive (28/28). However, one study reported 31 cases that were not included (22). This disease, caused by gene mutations, has been reported in Asian (3/135, 2.22%), European (26/135, 19.26%), and American (106/135, 78.52%) populations, with the majority of cases detected in Europe and America (Table 1) (including 31 cases involving American children). To date, only four patients with CSID have been reported in China, one of whom was reported to have compound heterozygous mutations in the *SI* gene (5, 21).

**Table 1:** Genotypic and phenotypic features of all reported patients with CSID and *SI* mutations.

	**Genotypic features**						**Clinical manifestations**				
**Case (reference)**	**Nt(AA) change (NM_001041.4)**	**Mutation type**	**Zygotic type**	**Domain of mutation**	**Geographical origin**	**Sex (F/M)**	**Diarrhea**	**Onset of diarrhea**	**Failure to thrive**	**Enzyme activities**	
										**Sucrase**	**Isomaltase**
						**19/60**	**29/29**		**28/28**		
Present	c.2626C > T (p.Q876*)	Non-sense	Compound heterozygote	Isomaltase	Asia	M	+	3 months	+	NA	NA
	c.2872C > T (p.R958C)	Missense		Isomaltase						NA	NA
Marcadier et al. (16)	c.273_274delAG (p.Gly92Leufs*8)	Frameshift	Homozygote	Stalk	America	F	+	9 days	+	NA	NA
Gericke et al. (6)	c.315G > T (p.Trp105Cys)	Missense	compound heterozygote	Stalk	Europe	NA	+	NA	+	Reduced	Reduced
	p.Trp931*	Non-sense		Isomaltase						Inactive	Inactive
Spodsberg et al. (13)	c.350A > G (p.Gln117Arg)	Missense	Homozygote	Isomaltase	Europe	NA	+	NA	+	Reduced	Reduced
Gericke et al. (6)	c.416T > A (Phe139Tyr)	Missense	NA	Isomaltase	Europe	NA	+	NA	+	Normal	Normal
Capalbo et al. (17)	c.853G > T (p.Glu285*)	Non-sense	Heterozygote	Isomaltase	America	F	NA	NA	NA	NA	NA
Capalbo et al. (17)	c.853G > T (p.Glu285*)	Non-sense	Heterozygote	Isomaltase	America	M	NA	NA	NA	NA	NA
Gericke et al. (6)	p.Gln307Try	Missense	NA	Isomaltase	Europe	NA	+	NA	+	Normal	Reduced
Jacob et al. (12)	c.1021T > C (p.Leu340Pro)	Missense	Homozygote	Isomaltase	Europe	NA	+	NA	+	Normal	Normal
Gericke et al. (6)	c.1607A > T (p.Asp536Val)	Missense	compound heterozygote	Isomaltase	Europe	NA	+	NA	+	Reduced	Inactive
	c.1730T > G (p.Val577Gly)	Missense		Isomaltase						Inactive	Inactive
Sander et al. (2)	c.1648delC	Frameshift	compound heterozygote	Isomaltase	Europe	M	+	NA	+	NA	NA
	c.4099A > G (p.Arg1367Gly)	Missense		Sucrase						NA	NA
Sander et al. (2)	c.26887+1G > C	Splicing	compound heterozygote	Isomaltase	Europe	NA	+	NA	+	Inactive	Inactive
	c.1780T > C (p.Ser594Pro)	Missense		Isomaltase						Inactive	Inactive
Sander et al. (2)	c.26887+1G > C	Splicing	compound heterozygote	Isomaltase	Europe	NA	+	NA	+	Inactive	Inactive
	c.1780T > C (p.Ser594Pro)	Missense		Isomaltase						Inactive	Inactive
Sander et al. (2)	c.1730T > G (p.Val577Gly)	Missense	compound heterozygote	Isomaltase	Europe	M	+	NA	+	Inactive	Inactive
	c.3218G > A (p.Gly1073Asp)	Missense		Sucrase						Inactive	Inactive
Sander et al. (2)	c.1730T > G (p.Val577Gly)	Missense	compound heterozygote	Isomaltase	Europe	M	+	NA	+	Inactive	Inactive
	c.3218G > A(p.Gly1073Asp)	Missense		Sucrase						Inactive	Inactive
Capalbo et al. (17)	c.1730T > G (p.Val577Gly)	Missense	Heterozygote	Isomaltase	America	F(5cases)	NA	NA	NA	NA	NA
Capalbo et al. (17)	c.1730T > G (p.Val577Gly)	Missense	Heterozygote	Isomaltase	America	M(18cases)	NA	NA	NA	NA	NA
Ceyhann-Birsoy et al. (18)	c.1730T > G (p.Val577Gly)	Missense	NA	Isomaltase	America	NA	NA	NA	NA	NA	NA
Gericke et al. (6)	c.1780T > C (p.Ser594Pro)	Missense	NA	Isomaltase	Europe	NA	+	NA	+	NA	NA
Ritz et al. (14)	c.1859T > C (p.Leu620Pro)	Missense	Homozygote	Isomaltase	Europe	NA	+	NA	+	Inactive	Inactive
Keiser et al. (15)	c.1903T > C (p.Cys635Arg)	Missense	Homozygote	Isomaltase	Europe	M	+	NA	+	Reduced	Reduced
Hou et al. (20)	c.1936delT (p.Cys646fs)	Frameshift	Heterozygote	Isomaltase	America	NA	NA	NA	NA	NA	NA
Sander et al. (2)	c.2080A > C (p.Thr694Pro)	Missense	Heterozygote	Isomaltase	Europe	NA	+	NA	+	NA	NA
Ceyhann-Birsoy et al. (18)	c.2159+2T > G	Splicing	NA	Isomaltase	America	NA	NA	NA	NA	NA	NA
Gericke et al. (6)	p.Leu741Pro	Missense	compound heterozygote	Isomaltase	Europe	NA	+	NA	+	Inactive	Inactive
	c.5234T > G (p.Phe1745Cys)	Missense		Sucrase						Inactive	Inactive
Wang et al. (21)	c.2311delA	Frameshift	compound heterozygote	Isomaltase	Asia	F	+	NA	NA	NA	NA
	c.5056C > T (p.Arg1686Cys)	Missense		Sucrase						NA	NA
Cheema et al. (19)	c.2401G > T (p.Glu801*)	Non-sense	NA	Isomaltase	Asia	NA	NA	NA	NA	NA	NA
Gericke et al. (6)	c.2789A > G (p.Gln930Arg)	Missense	compound heterozygote	Isomaltase	Europe	NA	+	NA	+	Normal	Normal
	p.Arg1544Cys	Missense		Sucrase						Reduced	Reduced
Gericke et al. (6)	p.Trp931Arg	Missense	compound heterozygote	Isomaltase	Europe	NA	+	NA	+	Reduced	Reduced
	p.Thr1606Ile	Missense		Sucrase						Reduced	Reduced
Capalbo et al. (17)	c.3186_3187delTT (p.Tyr1063fs)	Frameshift	Heterozygote	Sucrase	America	F	NA	NA	NA	NA	NA
Capalbo et al. (17)	c.3186_3187delTT (p.Tyr1063fs)	Frameshift	Heterozygote	Sucrase	America	M (8cases)	NA	NA	NA	NA	NA
Sander et al. (2)	c.3218G > A (p.Gly1073Asp)	Missense	Heterozygote	Sucrase	Europe	NA	+	NA	+	Inactive	Inactive
Capalbo et al. (17)	c.3218G > A(p.Gly1073Asp)	Missense	Heterozygote	Sucrase	America	F (4cases)	NA	NA	NA	NA	NA
Capalbo et al. (17)	c.3218G > A(p.Gly1073Asp)	Missense	Heterozygote	Sucrase	America	M (18cases)	NA	NA	NA	NA	NA
Ceyhann-Birsoy et al. (18)	c.3218G > A(p.Gly1073Asp)	Missense	NA	Sucrase	America	NA	NA	NA	NA	NA	NA
Hou et al. (20)	c.3229C > T (p.Arg1077*)	Non-sense	Heterozygote	Sucrase	America	NA	NA	NA	NA	NA	NA
Hou et al. (20)	c.3266G > A (p.Trp1089*)	Non-sense	Heterozygote	Sucrase	America	NA	NA	NA	NA	NA	NA
Ouwendijk et al. (8)	c.3293A > C(p.Gln1098Pro)	Missense	Homozygote	Sucrase	Europe	NA	+	NA	+	Inactive	Inactive
Capalbo et al. (17)	c.3370C > T (p.Arg1124*)	Non-sense	Heterozygote	Sucrase	America	F (2cases)	NA	NA	NA	NA	NA
Capalbo et al. (17)	c.3370C > T (p.Arg1124*)	Non-sense	Heterozygote	Sucrase	America	M (6cases)	NA	NA	NA	NA	NA
Gericke et al. (6)	c.3370C > T (p.Arg1124*)	Non-sense	compound heterozygote	Sucrase	Europe	NA	+	NA	+	Inactive	Inactive
	c.3218G > A (p.Gly1073Asp)	Missense		Sucrase						Inactive	Inactive
Capalbo et al. (17)	c.3586_3587delAT (p.Met1196fs)	Frameshift	Heterozygote	Sucrase	America	F	NA	NA	NA	NA	NA
Capalbo et al. (17)	c.3586_3587delAT (p.Met1196fs)	Frameshift	Heterozygote	Sucrase	America	M (2cases)	NA	NA	NA	NA	NA
Sander et al. (2)	c.3686G > A(p.Cys1229Tyr)	Missense	compound heterozygote	Sucrase	Europe	F	+	NA	+	Inactive	Reduced
	c.5234T > G(p.Phe1745Cys)	Missense		Sucrase						Inactive	Inactive
Sander et al. (2)	c.3686G > A(p.Cys1229Tyr)	Missense	Heterozygote	Sucrase	Europe	F	+	NA	+	Inactive	Reduced
Naim et al. (7)	c.4427G > C (p.Gly1476Ala)	Missense	NA	Sucrase	NA	NA	NA	NA	NA	NA	NA
Gericke et al. (6)	c.4592G > A (p.Cys1531Tyr)	Missense	compound heterozygote	Sucrase	Europe	NA	+	NA	+	Inactive	Reduced
	c.3218G > A (p.Gly1073Asp)	Missense		Sucrase						Inactive	Inactive
Haberman et al. (11)	c.4593T > G (p.Cys1531Trp)	Missense	compound heterozygote	Sucrase	Asia	NA	NA	NA	NA	NA	NA
	c.1730T > G (p.Val577Gly)	Missense		Isomaltase						NA	NA
Capalbo et al. (17)	c.5110C > T (p.Arg1704*)	Non-sense	Heterozygote	Sucrase	America	F	NA	NA	NA	NA	NA
Sander et al. (2)	c.5234T > G(p.Phe1745Cys)	Missense	Heterozygote	Sucrase	Europe	M	+	NA	+	Inactive	Inactive
Sander et al. (2)	c.5234T > G(p.Phe1745Cys)	Missense	Heterozygote	Sucrase	Europe	M	+	NA	+	Inactive	Inactive

*F, female; M, male; CSID, Congenital sucrase-isomaltase deficiency; SI, sucrase-isomaltase; +, symptomatic; NA, Not available. The red shows the current cases*.

**Figure 3:**
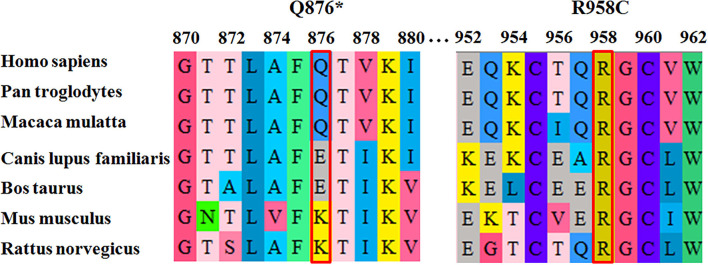
Conservation is shown in the red boxes; the Q876* mutated amino acids are moderately conserved across different species, and the R958C mutated amino acids are highly conserved.

Homozygous or compound heterozygous mutations in the *SI* gene were found *via* genetic testing to have caused CSID in our patient (7). We performed WES in this patient to confirm the diagnosis of CSID. WES identified novel compound heterozygous variants (c.2626C > T and c.2872C > T) in the *SI* gene. The p.Q876^*^ mutation can be interpreted as “likely pathogenic” according to the American College of Medical Genetics and Genomics (ACMG) standard, as this mutation is a null variant (pathogenic criterion PVS1) that is absent from controls (PM1) (23). The other variant, p.R958C, can also be classified as likely pathogenic, since this variant has an extremely low frequency in controls (PM1) and was detected in trans with another likely pathogenic variant (PM3). This variant was also predicted by multiple lines of prediction software to be deleterious (PM): it was predicted to be “deleterious” by PROVEAN, with a score of 6.45 (24); predicted to be “damaging” by SIFT, with a score of 0.000 (http://sift.bii.a-star.edu.sg); and predicted to be “probably damaging” by polyphen2, with a score of 1.0 (http://genetics.bwh.harvard.edu/pph2). The phenotype of the patient was also specific to the disease (PP4). In addition, residue R958 is highly conserved across various species (Figure 3), which indicates the functional importance of this residue. Thus, the changes in residue properties may damage the structure and function of the final product. We considered that both of these phenotypes were disease-causing mutations.

The *SI* gene encodes a protein of 1,827 amino acids that has four membrane-spanning regions (membrane anchor, stalk region, isomaltase domain, and sucrase domain); this protein is preferentially expressed in the small intestinal microvillus membrane, performing terminal digestion of dietary sucrose and starch (7). The two mutations in our report were located in the isomaltase domain from residues 110 to 1,007; in previous reports, there were two mutations reported in the stalk region, 19 in the isomaltase domain and 17 in the sucrase domain (Figure 2C). The first null variant p.Q876^*^ leads to a truncated protein with only 876 amino acids. Parts of the isomaltase domain and the whole sucrase domain are missing. All of the active sites in the sucrase domain, including residues 1,231, 1,259, 1,260, 1,295, 1,335, 1,393, 1,394, 1,395, 1,484, 1,497, 1,500, 1,533, and 1,558, are missing, which might seriously disrupt the function of the enzyme sucrase-isomaltase (25). The other mutation, p.R958C, is located in the trefoil factor domain from residues 935 to 980 of the isomaltase domain (https://www.ncbi.nlm.nih.gov/protein/NP_001032.2). This domain is highly expressed by mucus-producing cells and is thought to be related to mucosal defense (26).

According to the database Human Gene Mutation Database (HGMD) Professional, 40 mutations in the *SI* gene have been identified as associated with CSID (Table 1). The reported mutations included missense (25/40, 62.5%), non-sense (7/40, 17.5%), andFrameshiftmutations (6/40, 15.0%) and mutations at the splice site (2/40, 5.0%). Three zygotic mutations have also been reported, including homozygotes (6/97, 6.19%), compound heterozygotes (15/97, 15.46%), and one case of heterozygotes (76/97, 78.35%). Because details are lacking, the data from 31 American children are not included in the above summary.

Although we did not detect sucrase and/or isomaltase activity in biopsy tissue of the small bowel, most biopsies in the reported cases revealed reduced or absent enzyme activities (sucrase and isomaltase) (Table 1). Fortunately, after 3 years of follow-up, the patient in this report gradually grew under a strict sucrose- and starch-restricted diet. Therefore, lifelong sucrose restriction is an effective therapy for patients with CSID (7).

In conclusion, the clinical manifestations, genetic results, and effective treatment support our diagnosis. Without diagnosis, the treatment would not have been appropriate, and the boy may have continued to have diarrhea and failure to thrive. If endoscopy is not allowed, genetic evaluation with WES can be used as a diagnostic tool. Due to the development of the field of genetics, we were able to describe a novel case with mutations in the *SI* gene that caused CSID, which provides valuable information for the clinical field, especially in China.

## Data Availability Statement

The original contributions presented in the study are included in the article/Supplementary Material, further inquiries can be directed to the corresponding author/s.

## Ethics Statement

The studies involving human participants were reviewed and approved by Shenzhen Children's Hospital. Written informed consent to participate in this study was provided by the participant's legal guardian/next of kin.

## Author Contributions

JZ, MC, and SZ: conceptualization and writing—original draft. YZ and XQ: data collection. YZ, XQ, YC, and HC: formal analysis. SZ: funding acquisition and project administration. JZ: investigation. JZ, XQ, HC, and SZ: writing—review and editing. All authors have read and approved the manuscript.

## Funding

This work was supported by the Science Technology and Innovation Committee of Shenzhen (2021N062-JCYJ20210324115408023) and supported by Shenzhen Fund for Guangdong Provincial High level Clinical Key Specialties (No. SZGSP012).

## Conflict of Interest

The authors declare that the research was conducted in the absence of any commercial or financial relationships that could be construed as a potential conflict of interest.

## Publisher's Note

All claims expressed in this article are solely those of the authors and do not necessarily represent those of their affiliated organizations, or those of the publisher, the editors and the reviewers. Any product that may be evaluated in this article, or claim that may be made by its manufacturer, is not guaranteed or endorsed by the publisher.

## Abbreviations

CSID: congenital sucrase-isomaltase deficiency
SI: sucrase-isomaltase
kg: kilogram
IgE: immunoglobulin E
ACMG: American College of Medical Genetics and Genomics.
